# Detection of physiological changes in knee cartilage using parametric T2 relaxation maps estimated with a dictionary method

**DOI:** 10.1007/s00421-025-05898-9

**Published:** 2025-08-23

**Authors:** José M. Coelho, Tiago T. Fernandes, Sandra M. Alves, Adélio Vilaça, Rita G. Nunes, Luísa Nogueira, António Oliveira

**Affiliations:** 1 Department of Radiology, Centro Hospitalar Universitário de Santo António (CHUdSA), Unidade Local de Saúde de Santo António, Clínica de Imagiologia Diagnóstica E de Intervenção, Porto, Portugal; 2 Radiology Department, Escola Superior de Saúde/Instituto Politécnico do Porto, Rua António Bernardino de Almeida,400, 4200-072 Porto, Portugal; 3 Institute for Systems and Robotics—Lisboa and Department of Bioengineering, Instituto Superior Técnico, Universidade de Lisboa, Lisbon, Portugal; 4 Escola Superior de Saúde/Instituto Politécnico do Porto, Rua António Bernardino de Almeida, 400, 4200-072 Porto, Portugal; 5 Centre for Health Studies and Research of the University of Coimbra/Centre for Innovative Biomedicine and Biotechnology (CEISUC/CIBB), Coimbra, Portugal; 6 REQUIMTE/LAQV, ESS, Polytechnic of Porto, Rua Dr. António Bernardino de Almeida, 400, 4200-072 Porto, Portugal; 7 Orthopedic Department, Centro Hospitalar Universitário de Santo António (CHUdSA), Unidade Local de Saúde de Santo António, Porto, Portugal; 8 Unidade Multidisciplinar de Investigação Biomédica (UMIB), ICBAS, School of Medicine and Biomedical Sciences, University of Porto, Porto, Portugal; 9 ITR-Laboratory for Integrative and Translational Research in Population Health, Porto, Portugal; 10 EPIUnit-Instituto de Saúde Pública, Universidade do Porto, Porto, Portugal; 11 ICBAS, School of Medicine and Biomedical Sciences, University of Porto, Porto, Portugal

**Keywords:** Knee articular cartilage, T2 mapping, Dictionary matching, Running, Recovery, Overtraining syndrome

## Abstract

**Purpose:**

To investigate half-marathon running and recovery effects on knee articular cartilage (KAC) health in athletes, using dictionary-matching T_2_ maps, to detect changes and recovery patterns in KAC.

**Methods:**

Eleven asymptomatic volunteers (4 females, 7 males; mean age 40 ± 5 years, mean BMI 22.7 ± 1.4 kg/m^2^) completed a fixed-pace half-marathonwere studied. All were right-knee dominant and engaged in regular running. Sagittal T_2_-weighted Multi-Echo Spin-Echo images at 3 T were used to assess T_2_ values pre-run, post-run, and one week later for global and compartmental KAC. Recovery programme included low-intensity running, strength training and rest. Repeated measures ANOVA or equivalent non-parametric tests with post-hoc comparisons compared T_2_ values over time. Separate analyses were conducted based on laterality, gender and anatomical compartments. Significance was set at < 0.05.

**Results:**

Post-run T_2_ values decreased significantly by 0.9 ms (− 2.0%, p < 0.001), with up to 5.3% reductions in medial tibial (MT) and femoral (MF) compartments. Recovery patterns varied by compartment, sex and laterality. Most compartments returned to baseline within one week; the lateral condyle (LatC) showed incomplete recovery (− 4.7%, Proportional Recovery Index (PRI) = − 0,1); the right knee’s MT exhibited overcompensation (6.3%, PRI = 2.6). Males showed higher baseline T_2_ values and more efficient recovery in the LatC compared to females (PRI = − 0.1 vs. − 0.4).

**Conclusion:**

Half-marathon running induces reversible reductions in KAC hydration, with most compartments recovering or overcompensating within one week. Dictionary-matching T2 mapping offers a robust approach for monitoring cartilage integrity and guiding individualised recovery strategies.

**Supplementary Information:**

The online version contains supplementary material available at 10.1007/s00421-025-05898-9.

## Introduction

Sports participation is associated with increased knee articular cartilage (KAC) injuries, with chondral lesions observed in approximately 69.6% of knee arthroscopies for pain or instability, particularly in the medial femoral (MF) condyle (Mateos-Fernández et al. 2023). Moderate physical activity enhances cartilage resilience through collagen synthesis, proteoglycan production, and synovial fluid diffusion, enhancing mechanical resilience (Smith 2020; Hernández-Hermoso et al. 2021). However, endurance sports such as long-distance running impose repetitive mechanical stress that may exceed adaptative thresholds, increasing the risk of overuse-related lesions. These primarily include focal chondral defects and early signs of osteoarthritis (OA) (Ma et al. 2024), as well as meniscal tears and tendinopathies (Luca et al. 2022). Subclinical cartilage changes, reflect these biomechanical adaptations and may signal vulnerability to overuse in asymptomatic runners (Sandmeier 2000). While acute biochemical changes typically resolve within days in healthy athletes, indicating adaptive rather than degenerative responses (Coburn et al. 2023a), sustained alterations could unmask deficits in conditioning or recovery protocols, highlighting the need for personalized training strategies in endurance sports. Factors such as overtraining with inadequate recovery, poor running form, inadequate footwear, and pre-existing conditions can contribute to the risk of injuries. Insufficient rest periods can trigger overtraining syndrome (OTS), a state of prolonged imbalance between training load and recovery, characterised by systemic dysfunction including decreased performance, muscle weakness, pain, and psychological distress (Cadegiani 2020; Haghighat and Stull 2022). While OTS is traditionally associated with neuromuscular and metabolic dysregulation, its relationship to  KAC health is not fully understood, and there is no agreement on the appropriate KAC recovery time after prolonged efforts (Coburn et al. 2023b). Repeated cycles of mechanical stress without adequate recovery may overcome cartilage repair mechanisms, leading to cumulative microtrauma detectable via quantitative T2 MRI methods as incomplete recovery (e.g., residual T2 reductions). While acute post-exercise biochemical changes (e.g., transient T2 alterations) are part of adaptive remodelling, their persistence beyond typical recovery windows could signal early OTS-related KAC degeneration. Athletes engaged in high-frequency training are particularly vulnerable, as repetitive loading without sufficient rest may compound subclinical damage into structural pathology. For instance, prolonged insufficient recovery after acute mechanical stress (e.g., a half-marathon) may limit KAC repair, allowing transient microtrauma to evolve into chronic degeneration detectable via advanced imaging biomarkers. Post-run studies have shown transient KAC T_2_ value elevations after a half-marathon (Zhang et al. 2022; Coburn et al. 2023a, b), with persistent T_1rho_ and T_2_ changes post-marathon lasting up to 3 months, particularly in the patellofemoral joint and medial compartment (Luke et al. 2010),  suggest that inadequate recovery after repetitive efforts could evolve accumulate into chronic damage. Here, we focus on the one-week recovery window to determine whether T2 normalization correlates with structured rehabilitation, thereby mitigating OTS risks.

Conventional Magnetic Resonance Imaging (MRI) sequences lack the sensitivity to detect early physiological changes. Typically T_2_ T2 maps are generated by recording the signal decaywith T_2_-weighted multi-echo spin-echo (MESE) sequences measures T_2_ relaxation times and is a validated image KAC biomarker that correlates with the collagen structure and the water content, with high T2 values reflecting increased KAC hydration (Mosher and Dardzinski 2004). Typically, T_2_ maps are generated by recording the signal decay on a pixel-by-pixel basis and fitting a mono-exponential decay curve to estimate T_2_ values. However, this approach presents some limitations, namely sensitivity to diffusion effects, inaccuracies from stimulated echoes causing deviations of the signal decay from the mono-exponential model, and artefacts associated with the use of fast spin-echo sequences (Koff et al. 2008). Mono-exponential T2 mapping studies show that long-distance running induces immediate, often transient, T2 changes, reflecting post-run alterations in cartilage hydration (Mosher et al. 2005; Luke et al. 2010; Crowder et al. 2021; Khan et al. 2022; Coburn et al. 2023a). Moderate-distance running (6-20 km/week) correlates with healthier cartilage in frequent runners compared to non-runners or long-distance runners (Jandacka et al. 2024). However, demographic factors, like age and sex, may influence these effects with middle-aged individuals and women demonstrating higher baseline T2 values, suggesting possible structural KAC changes (Jandacka et al. 2024). Although changes greater than 5% in T2 values have been considered clinically meaningful in early symptomatic OA knees (Williams et al. 2025), direct equivalence to degenerative disease remains to be established and the limitations of the mono-exponential methods may still underestimate or misclassify such observations due to measurement errors. Dictionary-based methods overcomes these limitations by matching MESE signals to pre-calculated echo-modulation curves (EMC). This approach considers the shape of the radiofrequency pulses used in the protocol and the MESE acquisition parameters to generate a dictionary of theoretical decay curves, each associated with a unique T_2_ value and the transmit B1 + field. The optimal correlation between the recorded MESE signal and the pre-calculated EMC is determined to ascertain the T_2_ value for each pixel, enhancing accuracy over conventional mono-exponential fitting (Ben-Eliezer et al. 2015). Research has shown that EMC-T2 methods reduce T2 estimation errors to 1–11% compared to mono-exponential techniques (Raya et al. 2010; Haoyang et al. 2024), with sensitivity to detect physiological hydration changes as small as 0.61 ms (Coelho et al. 2025). This enhanced precision enables detection of subtle T2 alterations clinically relevant for early OA, where minor cartilage compositional changes precede structural damage.

EMC-T_2_ maps have also shown sensitivity to detect running-related KAC changes. In healthy adolescent basketball players, a 30 min, 5.77 km run induced changes in the central/posterior areas of the MF and the posterior MT, with no significant alterations in the lateral areas (Chechik et al. 2021). Post-meniscectomy individuals exhibited reduced post-run T_2_ values, especially in LF and LT, which were not observed in healthy knees, suggesting that the surgical history can also influence after-running recovery (Lindner et al. 2022).

This study aims to evaluate the recovery kinetics of KAC following a fixed-pace half-marathon distance run (21 km), using dictionary-based T2 mapping to identify delayed or incomplete recovery patterns that may reflect inadequate KAC recovery. To the best of our knowledge, this is the first study to use cartilage-specific recovery metrics with dictionary-matching T2 maps as biomarkers for monitoring OTS risk in endurance athletes. 

## Materials and methods

### Volunteer recruitment

This study, conducted from July/2024 to February/2025, received ethical approval from the Institutional Ethics Committee (2018203 (178-DEFI/177-CES)); recruited semi-professional athletes (training 5–7 days/week and periodically competing in regional half-marathons or marathons), with written informed consent; aged 18–50 years; of any ethnicity; who met the inclusion criterion of a Knee Injury and Osteoarthritis Outcome Score (KOOS) (Roos et al. 1998) Sport and Recreation (Sport/Rec) subscale score above 90 to exclude individuals with significant knee dysfunction (Kim 2023; Paris et al. 2024). The KOOS questionnaire, which evaluates knee-related symptoms and function capacity across 5 subscales (Pain, other Symptoms, Activities of Daily Living, Sports and Recreation, and Quality of Life) was used to screen for prior knee pathologies. Participants were also screened for knee injuries over the preceding 12 months via a supplementary questionnaire with none reporting prior trauma or surgery. Exclusion criteria included incomplete informed consent; pregnancy; MRI contraindications; signal alterations detected on the MRI images; and pre-existing knee, hip or spine pathologies identified by KOOS.

Demographic data (age, height, weight, body mass index (BMI), biological sex (male/female) and knee dominance) was collected, with BMI classified as “normal” (18.5 and 24.9 kg/m^2^); “above” (25.0 and 29.9 kg/m^2^); and “overweight” (above 30 kg/m^2^) (Weir and Jan 2024). All participants were right-knee dominant with running experience ranging from 10 to 20 years and weekly mileage ranging from 40 to 50 km.

### Exercise protocol

Participants followed a fixed gender-adapted training programme, before and after the half-marathon run (Table 1). Week 1  included a routine training comprised of 3 fixed-intensity runs, muscle strengthening sessions, and a rest day prior to the data acquisition. The last day included a rest MRI exam (labelled “rest”), a fixed-pace half-marathon (21 km), and a post-run MRI exam (labelled “21k”). The 21 km run was conducted on an urban street route (completion time: ~1h45min males, ~2h07min females), with a fixed pace (males: 5’00”/km ±5”; females: 6’00”/km ±5") to ensure consistent mechanical stress. Pacing was used as the primary intensity metric. MRI scans (rest, 21k, control) were performed almost immediately upon arrival, with no rest period. Post-run MRI commenced within 5 minutes of race completion to capture acute cartilage changes. Week 2 focused on recovery, involving supervised lower-body resistance exercises (squats, lunges, leg presses) and core stability training at 70–80% of one-repetition maximum lasting 45–60 minutes per session. The control MRI exam (labelled “control”) was acquired one week post-race without standardized pre-scan rest.

**Table 1:** Weekly training program before each MRI exam

Days Activity		Monday	Tuesday	Wednesday	Thursday	Friday	Saturday	Sunday			Marathon time	Weekly run distance
		Endurance Run	Muscle Strengthening	Endurance Run	Rest	Light Run	Rest	Rest MRI	Half Marathon	21k MRI		
Week 1												
Distance Pace	Males	12 km 4′45"–5′00"/km	-	12 km 4′45"–5′00"/km	-	8 km 5′00"/km	-	-	21,1 km fixed: 5′00", maximum oscillation of 5"/Km	-	~1h 45 min	53.1 km
	Females	10 km 6′00"/km	-	10 km 6′00"/km	-	7 km 6′00"/km	-	-	21,1 km fixed: 6′00", maximum oscillation of 5"/Km	-	~2h 07 min	48.1 km

Days Activity		Monday	Tuesday	Wednesday	Thursday	Friday	Saturday	Sunday Control MRI			-	Weekly run distance
		Light Run	Muscle Strengthening	Tempo Run	Rest	Light Run	Rest					
Week 2												
Distance Pace	Males	6 km 5′20"–5′30"/km	-	10 km 4′45"–5′00"/km	-	12 km 4′45"–5′00"/km	-	-			-	28 km
	Females	5 km 6′20"–6′40"/km	-	8 km 6′00"/km	-	10 km 6′00"/km	-	-			-	23 km

The Proportional Recovery Index (PRI) (Goldsmith et al. 2022) was used to assess T_2_ value recovery as a measurable indicator of recovery efficiency. The PRI was calculated as the ratio between the recovery of the T_2_ values from 21 k to control, and the reduction of the T_2_ values from rest to 21 k. A PRI = 1 indicates complete T_2_ recovery to baseline values; PRI < 1 indicates incomplete T_2_ recovery, and a PRI > 1 suggests overcompensation of T_2_ recovery. A positive PRI would indicate that the biological processes responsible for rehydrating cartilage are functioning effectively. Inversely, negative PRR values suggest that cartilage dehydration has worsened over time, reflecting a lack of recovery or even further deterioration.

### MRI protocol

A total of 30 sagittal T_2_ KAC slices were obtained from both knees using a Philips Achieva 3 T MRI scanner (Achieva 3.0 T TX; Philips Medical Systems, Best, The Netherlands) with an 8 channel knee coil. Each participant lay supine, with the non-dominant knee scanned first to minimise the influence of knee dominance on gait force effects and KAC hydration (Wang and Watanabe 2012; Goto et al. 2022), placed on the antenna with a mean flexion of 10º.

An in vivo T_2_ MESE optimised sequence (Freitas et al. 2020) was used: TR of 2057 ms; 10 evenly spaced echoes (TE ranging from 5.9 ms—59 ms), with a step size of 5.9 ms; 2.5 mm slice thickness with no spacing; interleaved acquisition; 220 × 206 matrix; field of view 150 mm; 0.29 × 0.29 mm^2^ in-plane pixel resolution; bandwidth of 48.5 kHz; 1 signal average and a total scan time of 9:15 min. Scans were labelled by knee side and dominance (e.g., ‘dom_right’), and exercise phase (“rest”, “21 k” and “ctrl”, respectively).

All scans were acquired by the same researcher, with the sagittal plane oriented using standard anatomical landmarks to ensure consistent slice placement. This plane allows for a better KAC assessment in the direction perpendicular to most of the joint weight forces. Ink markings were applied on the skin surface over the anatomical bone landmark of the patella and tibial tuberosity during the first scan to minimise positioning variations in post-run scans.

### Image segmentation and quantitative analysis

The segmentation process was preceded by KAC inspection of the T_2_-weighted sagittal images, for detection of signal and morphological alterations. Image segmentation was performed using ITK-SNAP v4.0 (Yushkevich et al. 2006) by a single researcher (JC), blinded to the patient’s identity and exercise condition to ensure unbiased and objective analysis.

**Fig. 1:**
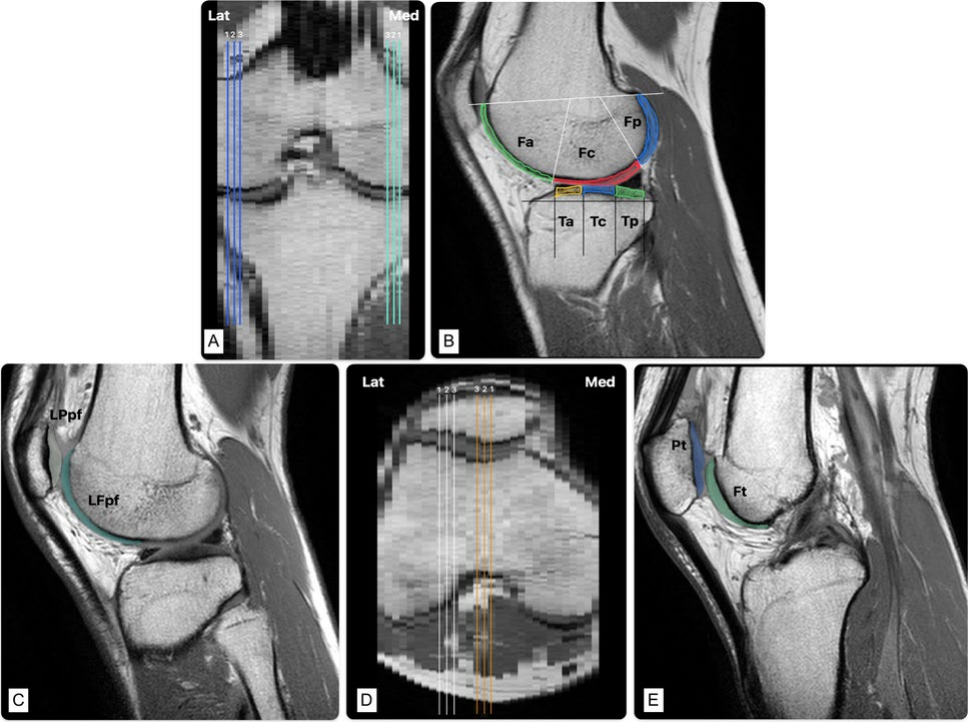
Slice ordering and KAC section definition. **A** and **D** Example of slice ordering on 3 contiguous central slices selected for segmentation of the knee cartilage, from the outermost to the innermost slice. **B** Whole-Organ Magnetic Resonance Imaging Score. Using the WORMS classification as a reference, the cartilage was subdivided in each sagittal image by its anatomical landmarks. The femoral articular surface was divided into medial condyle (MF) and lateral condyle (LF). Each condyle was further subdivided into three regions: anterior (a), central (c), and posterior (p). The medial tibial plateau (MT) and lateral tibial plateau (LT) are also divided into three regions: anterior (a), central (c), and posterior (p). **C** and **E** Example of the patellofemoral subdivisions. *Lat* lateral, *Med* medial, *Fa* femoral anterior, *Fc* femoral central, *Fp* femoral posterior, *Ta* tibial anterior, *Tc* tibial central, *Tp* tibial posterior, *LPpf* lateral patella of patellofemoral, *LFpf* lateral femur of patellofemoral, *Pt* patella of the trochlea, *Ft* femur of the trochlea. Source: investigator’s dataset

A semi-automatic two-step segmentation approach was employed, with the software’s “Adaptive Brush” tool (Coelho et al. 2025). The first echo image was used to differentiate deep cartilage from the underlying subchondral bone, while the last echo image was used to manually correct the initial segmentation by separating the superficial cartilage from the surrounding joint space. This process was applied to three consecutive central slices in four main KAC areas: medial and lateral condyles (LatC and MedC—including both femoral and tibial KAC in each), trochlear (TrPF) and lateral patellofemoral (LPF) cartilage. Condyle sagittal slices were chosen using the coronal and axial views to identify the most central slice in each condyle and select the lateral and medial contiguous slices. Subsequently, all three slices were ordered unequivocally from 1 to 3, orientated from the outermost to the innermost one (Fig. 1A and D). A similar method was used to select and order the central patellofemoral (PF) slices, using sagittal and axial views to ensure consistency. The central TrPF slice was identified in the axial view by selecting the slice with the most prominent trochlear angle and then choosing the sagittal slice located at the vertex of the trochlear angle. The central LPF slice was determined by measuring the length of the LPF KAC from the trochlear angle to the lateral limit of the articulation in the same axial view used before and defining the midpoint as the central sagittal cut.

The four main KAC areas were compartmentalised using the Whole-Organ Magnetic Resonance Imaging Score (Peterfy et al. 2004) as a reference (Fig. 1B). Femoral and tibial condyle cartilages were divided into 4 compartments on the sagittal plane: medial femoral (MF), lateral femoral (LF), medial tibial (MT) plateau and lateral tibial (LT) plateau; each was further divided into anterior, central and posterior regions. Meniscus margins defined the central weight-bearing femoral cartilage borders: anterior (“a”)—extended from the anterior peripheral meniscus margin to the anterior–superior osteochondral junction; central (“c”)—spanned from the anterior to the posterior meniscus margins; and posterior (“p”)—extended from the posterior meniscus margin of the to the posterior-superior osteochondral junction. Tibial cartilages were also divided into “a” (anterior), “c” (central), “p” (posterior) regions with “a” and “p” corresponding to meniscus-covered areas and “c” to the central weight-bearing region between meniscus horns. The PF cartilage was divided into (Fig. 1C–E) Lateral Patella of the PF (LPpf) and Lateral Femur of the PF (LFpf). These two compartments were considered representative of the LPF articulation. The trochlear zone was segmented into Trochlear Patella (Pt) and Trochlear Femur (Ft), combined as the TrPF articulation. Finally, one other compartment was defined: total T2 (T2Tot). Each slice was considered representative of one KAC sample (Chechik et al. 2021; Coelho et al. 2025), yielding 25 regions across 3 consecutive slices. Motion artefacts excluded data from three knees, leaving 19 knees and 57 valid cases completed for data analysis.

**Fig. 2:**
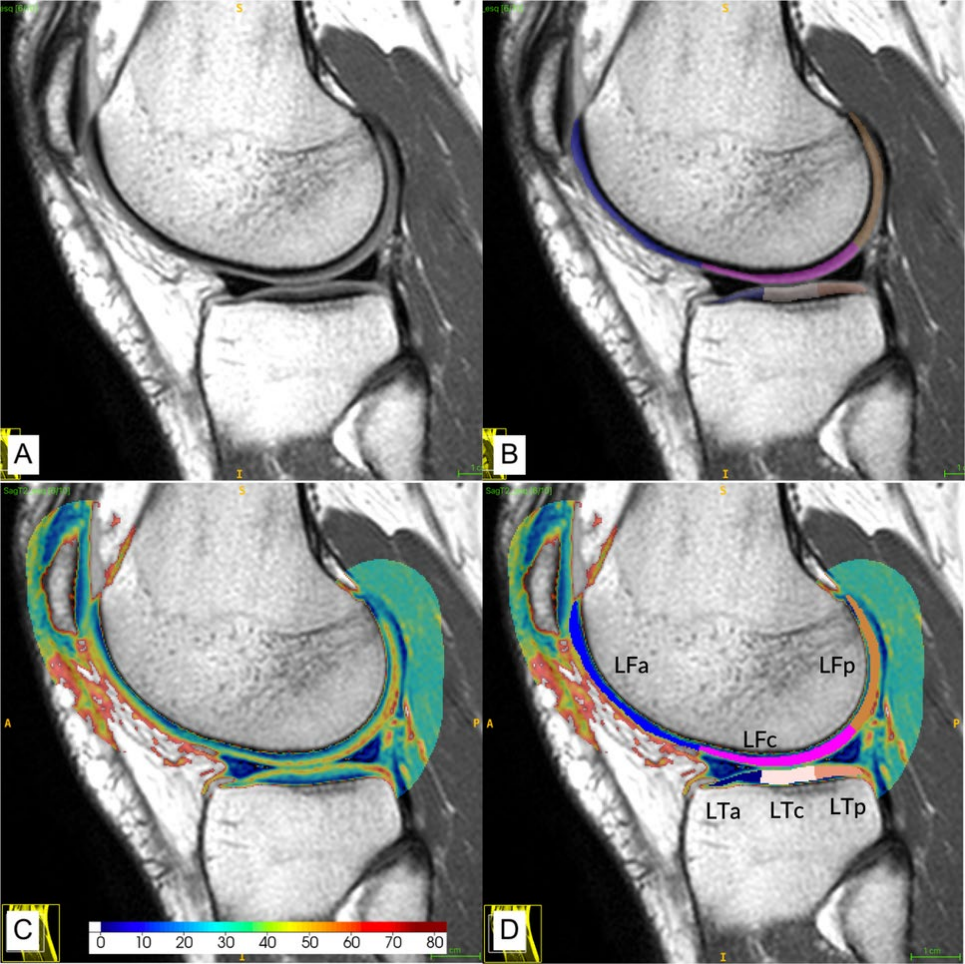
Example of KAC T2 measure in LatC. **A** LatC Sagittal MESE T2. **B** Segmentation of LF and LT (anterior, central, posterior). **C** Quantitative EMC T2 map superimposed on MESE T2 image. **D** KAC sections. Source: investigator’s dataset

Quantitative T_2_ maps were generated with the T2 KneeActive software tool available at (https://github.com/ZemaTimoteo/T2_KneeActive) (Fernandes et al. 2023). EMC matching involved creating EMC-dictionaries for T_2_ values ranging from 1 to 300 ms with a step size of 1 ms, T1 set at 1000 ms and B1 + values ranging from 60 to 140% with 1% increments (Mittal et al. 2019), matching the sequence parameters and RF pulse excitation/refocusing flip angles (90º/125º), and their shapes, as suggested by Ben-Eliezer (Ben-Eliezer et al. 2015). Template matching was performed by identifying the EMC with the maximum dot product with each pixel echo series. The map was processed pixel-wise, using the KAC segmentation mask. EMC-maps were superimposed on the last echo sagittal image, using the ITK-SNAP software, to manually refine the above-mentioned regions-of-interest (ROIs) by removing pixels with a T2 relaxation time greater than 100 ms corresponding to synovial liquid pixels (Fig. 2).

### Statistical analysis

The statistical analysis was performed with Microsoft Excel (version 16.72) and IBM SPSS Statistics (version 29.0). Mean T_2_ value (ms) and standard deviation were calculated for each KAC ROI, with 57 valid KAC samples with all three stages (rest, 21 k, control). Normality was assessed using Kolmogorov–Smirnov test, skewness and kurtosis (George and Mallery 2010). Repeated measures ANOVA compared the total T2 values across the 3 time points, with Mauchly´s test for sphericity and Bonferroni post-hoc corrections for specific comparisons (rest vs 21k and rest vs control). A similar analysis was stratified by laterality, biological sex and anatomical regions, therefore comparisons between subgroups (male vs. female, right vs. left knee) were conducted using descriptive statistical measures only. These included means, standard deviations, and percentage changes in T2 values across time points.. For anatomic regions and sections, Greenhouse–Geisser or Huynh–Feldt corrections addressed sphericity violations. Non-parametric Friedman tests with Bonferroni corrections were applied for normality violations. A p value lower than 0.05 was considered significant.

## Results

### Subject demographics

This study included both knees from 11 asymptomatic volunteers (4 female and 7 male) with a mean age of 40 ± 5 years. The mean BMI was 22.7 ± 1.4 kg/m^2^, placing all participants in the “normal” BMI category. All participants were right-knee dominant and participated in regular exercise and running programs. The database resulted in 57 KAC complete samples (42 male (73.7%) and 15 female (26.3%)).

### Immediate effects of half-marathon running on KAC T2 values

Post-run global T2 values (Table 2) showed a significant decrease of 0.9 ms (− 2.0%, p < 0.001). In some specific compartments (Table 3), significant post-run decreases were observed (p < 0.001), with some reductions exceeding 5%: MF (− 3.4%), MT (− 5.2%), LatC (− 4.1%), MedC (− 5.3%) and TrPF (− 2,7%).

**Table 2:** Global cartilage mean T2 values by knee laterality and sex

		N	T2 rest (ms)†	T2 21 k (ms)†	T2 control (ms)†	Difference rest → 21 k			Difference rest → control			PRI
						p value	(ms)†	(%)†	p value	(ms)†	(%)†	
Total T2		57	43.1 ± 2.6	42.2 ± 2.2	43.1 ± 2.7	< 0.001	− 0.9 ± 1.1	− 2.0 ± 2.5	~ 1	0.1 ± 1.4	0.2 ± 3.2	1.0
Laterality	Left	30	42.8 ± 2.8	41.9 ± 2.2	42.5 ± 2.7	0.002	− 0.9 ± 1.3	− 2.0 ± 3.0	0.847	− 0.3 ± 1.4	− 0.6 ± 3.4	0.8
	Right	27	43.4 ± 2.3	42.5 ± 2.2	43.8 ± 2.5	< 0.001	− 0.9 ± 0.8	− 2.1 ± 1.7	0.181	0.4 ± 1.2	1.0 ± 2.7	1.2
Sex	Male	42	43.6 ± 2.4	42.7 ± 2.0	43.7 ± 2.4	< 0.001	− 0.9 ± 1.0	− 2.0 ± 2.4	~ 1	0.0 ± 1.1	0.1 ± 2.5	1.1
	Female	15	41.5 ± 2.5	40.6 ± 1.9	41.6 ± 3.1	0.033	− 0.9 ± 1.2	− 2.1 ± 2.7	~ 1	0.1 ± 2.0	0.4 ± 4.7	1.1

**N** – number of samples (KAC slices); † - mean ± standard deviation; **Difference Rest → 21k**: T2 value loss in milliseconds caused by half-marathon running. **Difference Rest → Control**: T2 value recovery to baseline levels in milliseconds; **PRI** - Proportional Recovery Index. Detailed Information on ANOVA repeated measures statistics is given in supplementary table in Online Resource 1

**Table 3:** Cartilage mean T2 values by anatomic compartment

	T2 rest (ms)^a^	T2 21 k (ms)^a^	T2 control (ms)^a^	Difference Rest → 21 k			Difference rest → control			PRI
				p value	(ms)^a^	(%)^a^	p value	(ms)^a^	(%)^a^	
LF	45.0 ± 3.9	44.4 ± 3.4	45.0 ± 4.0	0.057	− 0.5 ± 1.7	− 1.1 ± 3.7	~ 1	0.0 ± 2.2	0.1 ± 4.9	1.0
LT	36.4 ± 5.4	36.9 ± 5.3	36.0 ± 5.4	0.458	0.6 ± 3.0	2.0 ± 8.1	~ 1	− 0.4 ± 3.0	− 0.7 ± 8.2	1.8
MF	45.9 ± 4.1	44.2 ± 3.3	45.3 ± 4.0	< 0.001	− 1.7 ± 2.2	− 3.4 ± 4.5	0.084	− 0.6 ± 2.0	− 1.2 ± 4.1	0.6
MT	40.8 ± 5.0	38.5 ± 4.1	41.4 ± 5.3	< 0.001	− 2.3 ± 3.3	− 5.2 ± 7.7	0.959	0.6 ± 4.6	2.1 ± 11.0	1.3
LatC	42.4 ± 3.0	40.7 ± 3.7	40.5 ± 4.2	< 0.001	− 1.8 ± 2.5	− 4.1 ± 5.9	0.001	− 2.0 ± 2.7	− 4.7 ± 6.5	− 0.1
MedC	43.7 ± 2.8	41.4 ± 3.3	43.4 ± 3.9	< 0.001	− 2.3 ± 1.9	− 5.3 ± 4.3	~ 1	− 0.3 ± 2.8	− 0.7 ± 6.3	0.9
LPF	44.2 ± 2.9	43.8 ± 3.0	44.4 ± 3.5	0.353	− 0.3 ± 2.8	− 0.4 ± 6.8	~ 1	0.4 ± 2.8	1.2 ± 7.0	1.5
TrPF	44.0 ± 2.9	42.8 ± 2.6	44.2 ± 2.8	< 0.001	− 1.2 ± 1.9	− 2.7 ± 4.2	0.930	0.2 ± 1.6	0.6 ± 3.8	1.2

Difference Rest→21 k: T2 value loss in milliseconds caused by half-marathon running. Difference Rest→Control: T2 value recovery to baseline levels in milliseconds. Detailed Information on ANOVA repeated measures statistics is given in supplementary table in Online Resource 2

*LF* Lateral Femur, *LT* Lateral Tibia, *MF* Medial Femur, *MT* Medial Tibia, *LatC* Lateral Condyle, *MedC* Medial Condyle, *LPF* Lateral Patellofemoral, *TrPF* Trochlea Patellofemoral, *PRI* Proportional Recovery Index

^a^Mean ± standard deviation

Section‐based analysis (Table 4) revealed that, within LF, the central (− 1.9%, p = 0.024) section decreased significantly; in MF, the anterior (− 2.6%, p = 0.026), central (− 2.3%, p = 0.012) and posterior (− 4.5%, p < 0.001) sections all exhibited significant reductions. All MT sections demonstrated significant decreases (anterior: − 5.0%, central: − 5.3%, posterior: − 4.6%; all p values < 0.05, 0.001), while the central section of the LT increased by + 5.4% (p = 0.017). Additionally, the TrPF T2 values decreased by 2.5% (Pt) and 2.7% (Ft) (both p < 0.05).

**Table 4:** Cartilage mean T2 values by section in each anatomic compartment

Compartments	Sections	T2 rest (ms)^a^	T2 21 K (ms)^a^	T2 control (ms)^a^	Difference rest → 21 k			Difference Rest → control			PRI
					p value	(ms)^a^	(%)^a^	p value	(ms)^a^	(%)^a^	
LF	Anterior	45.8 ± 5.8	45.7 ± 5.7	45.9 ± 5.5	*ns*	− 0.1 ± 3.8	0.2 ± 8.3	*ns*	0.1 ± 4.2	0.7 ± 4.2	2.0
	Central	42.7 ± 4.0	42.0 ± 4.2	42.4 ± 4.4	0.024	− 0.8 ± 2.3	− 1.9 ± 5.6	0.491	− 0.5 ± 2.5	− 1.0 ± 5.8	0.6
	Posterior	46.3 ± 4.5	45.6 ± 4.0	46.6 ± 4.3	0.106	− 0.7 ± 2.5	− 1.3 ± 5.2	0.872	0.3 ± 2.2	0.8 ± 4.7	1.4
LT	Anterior	35.4 ± 6.2	35.9 ± 6.4	36.1 ± 6.6	*ns*	0.6 ± 4.1	2.2 ± 12.0	*ns*	0.7 ± 4.1	2.7 ± 11.9	− 0.4
	Central	31.0 ± 5.7	32.5 ± 6.0	30.5 ± 5.6	0.017	1.5 ± 3.9	5.4 ± 12.3	0.603	− 0.5 ± 3.1	− 1.3 ± 9.8	1.3
	Posterior	42.7 ± 7.4	42.4 ± 5.9	41.4 ± 7.6	~ 1	− 0.4 ± 4.6	0.3 ± 11.2	0.069	− 1.4 ± 4.4	− 2.8 ± 10.7	− 3.3
MF	Anterior	47.0 ± 7.6	45.3 ± 5.4	46.3 ± 6.4	0.026	− 1.8 ± 6.1	− 2.6 ± 11.5	~ 1	− 0.8 ± 5.2	− 1.0 ± 9.1	0.6
	Central	40.7 ± 4.6	39.7 ± 4.9	40.1 ± 4.9	0.012	− 1 ± 2.4	− 2.3 ± 6.0	0.239	− 0.6 ± 2.5	− 1.4 ± 6.1	0.4
	Posterior	49.9 ± 3.9	47.6 ± 3.5	49.5 ± 3.9	< 0.001	− 2.3 ± 1.7	− 4.5 ± 3.2	0.248	− 0.4 ± 1.7	− 0.8 ± 3.3	0.8
MT	Anterior	44.0 ± 6.7	41.3 ± 6.0	45.5 ± 9.7	0.006	− 2.6 ± 6.2	− 5.0 ± 13.8	0.576	1.5 ± 8.6	4.1 ± 18.8	1.6
	Central	37.5 ± 5.7	35.1 ± 4.4	38.0 ± 6.3	< 0.001	− 2.4 ± 4.7	− 5.3 ± 11.7	~ 1	0.5 ± 6.0	2.3 ± 15.4	1.2
	Posterior	41.0 ± 5.8	39.1 ± 6.2	40.9 ± 6.1	0.003	− 2 ± 4.4	− 4.6 ± 10.6	~ 1	− 0.2 ± 3.6	− 0.2 ± 9.1	0.9
LPF	LPpf	41.4 ± 3.4	41.1 ± 2.4	41.8 ± 3.3	*ns*	− 0.3 ± 2.8	− 0.4 ± 6.8	*ns*	0.4 ± 2.8	1.2 ± 7.0	2.3
	LFpf	47.0 ± 5.1	46.4 ± 5.4	47.0 ± 5.9	*ns*	− 0.5 ± 3.2	− 1.0 ± 6.9	*ns*	− 0.0 ± 2.7	− 0.0 ± 5.9	1.0
TrPF	Pt	43.4 ± 3.2	42.2 ± 2.8	43.7 ± 3.2	0.007	− 1.2 ± 2.8	− 2.5 ± 6.4	~ 1	0.2 ± 2.5	0.7 ± 5.9	1.3
	Ft	44.6 ± 3.5	43.3 ± 3.2	44.8 ± 3.5	< 0.001	− 1.3 ± 2.0	− 2.7 ± 4.6	~ 1	0.2 ± 1.7	0.6 ± 3.9	1.2

*LF* Lateral Femur, *LT* Lateral Tibia, *MF* Medial Femur, *MT* Medial Tibia, *LatC* Lateral Condyle, *MedC* Medial Condyle, *LPF* Lateral Patellofemoral, *TrPF* Troclea Patellofemoral, *LPpf* Lateral Patella of the Patellofemoral, *LFpf* Lateral Femur of the Patellofemoral, *Pt* Patella of the Troclea, *Ft* Femur of the Troclea, *ns* not significant, *PRI* Proportional Recovery Index

^a^Mean ± standard deviation

Difference Rest→21 k: T2 value loss in milliseconds caused by half-marathon running; Difference Rest→Control: T2 value recovery to baseline levels in milliseconds; Detailed Information on ANOVA repeated measures statistics is given in supplementary table in Online Resource 3

### One week T2 recovery

After one week, global T2 values returned to baseline (+ 0.1 ms, 0.2%, p ~ 1, PRI = 1.0) (Table 2). Most compartments normalised (Table 3 and Table 4); however, LatC retained a significant residual decrease of − 2.0 ms (− 4.7%, p = 0.001, PRI = -0,1), suggesting no recovery in this region (Table 3 and Table 5). The left knee’s MedC showed minimal normalisation (− 2.9%, p = 0.019, PRI = 0.5), but above baseline. In the right knee, MT showed a recovery gain of 2.5 ± 4.6 ms (6.3%, p = 0.029, PRI = 2.6), suggesting overcompensation; while LatC showed minimal recovery with above baseline values: -2.8 ± 2.2 ms (− 6.4 ± 5.0%, p < 0.001, PRI = 0.1). Furthermore, in LatC, males (PRI = − 0.1, p < 0.001) recovered slightly better compared to females (PRI = − 0.4, p = 0.038).

**Table 5:** Laterality and Gender cartilage mean T2 values by anatomic compartment

Compartments	Laterality	T2 rest (ms)^a^	T2 21 K (ms)^a^	T2 control (ms)^a^	Difference rest → 21 k			Difference rest → control			PRI
	Sex				p value	(ms)^a^	(%)^a^	p value	(ms)^a^	(%)^a^	
LF	Left	44.6 ± 4.3	44.1 ± 3.6	44.4 ± 4.4	0.456	− 0.5 ± 1.8	− 0.9 ± 3.9	~ 1	− 0.2 ± 2.1	− 0.4 ± 4.9	0.6
	Right	45.4 ± 3.6	44.8 ± 3.3	45.6 ± 3.6	0.179	− 0.6 ± 1.6	− 1.2 ± 3.6	~ 1	0.2 ± 2.2	0.5 ± 5.1	1.3
	Male	46.0 ± 3.5	45.2 ± 3.1	45.7 ± 3.7	0.009	− 0.8 ± 1.6	− 1.6 ± 3.5	~ 1	− 0.3 ± 1.9	− 0.5 ± 4.1	0.6
	Female	42.2 ± 3.9	42.4 ± 3.6	42.8 ± 4.2	~ 1	0.2 ± 1.7	0.5 ± 4.0	~ 1	0.7 ± 2.8	1.7 ± 6.7	− 2.0
LT	Left	36.3 ± 6.3	38.2 ± 5.8	36.4 ± 5.8	< 0.001	1.9 ± 2.3	5.7 ± 6.6	~ 1	0.1 ± 2.1	0.9 ± 8.5	0.9
	Right	36.5 ± 4.3	35.6 ± 4.4	35.5 ± 5.0	0.399	− 0.9 ± 2.9	− 2.2 ± 7.7	0.285	− 0.9 ± 2.8	− 2.6 ± 7.7	− 0.1
	Male	35.9 ± 5.1	36.6 ± 5.1	36.0 ± 5.4	0.456	0.7 ± 3.0	2.3 ± 8.3	~ 1	0.0 ± 2.7	0.3 ± 7.7	0.9
	Female	37.6 ± 6.2	37.8 ± 5.9	36.0 ± 5.5	~ 1	0.2 ± 2.8	1.1 ± 7.9	0.313	− 1.6 ± 3.5	− 3.6 ± 9.3	9.0
MF	Left	46.3 ± 4.0	44.3 ± 3.1	45.5 ± 3.8	< 0.001	− 1.9 ± 2.5	− 3.9 ± 5.1	0.252	− 0.7 ± 2.2	− 1.4 ± 4.4	0.6
	Right	45.5 ± 4.3	44.1 ± 3.5	45.0 ± 4.3	< 0.001	− 1.4 ± 1.7	− 2.8 ± 3.7	0.568	− 0.5 ± 1.7	− 0.9 ± 3.8	0.6
	Male	46.7 ± 4.2	44.7 ± 3.2	46.0 ± 3.6	< 0.001	− 2.0 ± 2.3	− 4.0 ± 4.5	0.141	− 0.7 ± 2.1	− 1.3 ± 4.2	0.6
	Female	43.7 ± 3.2	42.9 ± 3.1	43.3 ± 4.4	0.326	− 0.8 ± 1.8	− 1.7 ± 4.2	~ 1	− 0.4 ± 1.7	− 1.0 ± 3.8	0.5
MT	Left	40.7 ± 5.5	37.8 ± 3.5	39.7 ± 4.1	< 0.001	− 2.9 ± 3.4	− 6.5 ± 7.6	0.485	-1.1 ± 4.0	− 1.8 ± 9.7	0.7
	Right	40.9 ± 4.6	39.3 ± 4.5	43.4 ± 5.9	0.025	− 1.7 ± 3.1	− 3.8 ± 7.7	0.029	2.5 ± 4.6	6.3 ± 11.0	2.6
	Male	42.1 ± 5.0	39.7 ± 3.7	43.1 ± 4.9	< 0.001	− 2.4 ± 3.4	− 5.1 ± 7.8	0.570	1.0 ± 5.1	3.2 ± 11.9	1.4
	Female	37.3 ± 3.3	35.1 ± 3.2	36.7 ± 3.0	0.041	− 2.2 ± 3.0	− 5.9 ± 7.6	~ 1	− 0.6 ± 2.8	− 1.3 ± 7.3	0.7
LatC	Left	41.7 ± 3.2	41.2 ± 4.0	40.4 ± 4.7	0.557	− 0.5 ± 2.1	− 1.3 ± 5.2	0.091	− 1.3 ± 3.0	− 3.1 ± 7.1	− 1.6
	Right	43.3 ± 2.6	40.2 ± 3.4	40.5 ± 3.6	< 0.001	− 3.1 ± 2.3	− 7.2 ± 5.1	< 0.001	− 2.8 ± 2.2	− 6.4 ± 5.0	0.1
	Male	42.7 ± 2.9	40.9 ± 3.5	40.8 ± 4.1	< 0.001	− 1.8 ± 2.5	− 4.2 ± 5.8	< 0.001	− 1.9 ± 2.3	− 4.4 ± 6.1	− 0.1
	Female	41.7 ± 3.2	40.1 ± 4.3	39.5 ± 4.6	0.091	− 1.6 ± 2.6	− 3.9 ± 6.5	0.038	− 2.3 ± 3.1	− 5.5 ± 7.7	− 0.4
MedC	Left	43.9 ± 3.1	41.1 ± 2.9	42.6 ± 3.6	< 0.001	− 2.8 ± 2.0	− 6.4 ± 4.5	0.019	− 1.3 ± 2.4	− 2.9 ± 5.5	0.5
	Right	43.4 ± 2.6	41.7 ± 3.7	44.2 ± 4.2	< 0.001	− 1.7 ± 1.5	− 4.1 ± 3.8	0.419	0.8 ± 2.7	1.7 ± 6.4	1.5
	Male	44.5 ± 2.5	42.2 ± 3.1	44.6 ± 3.5	< 0.001	− 2.3 ± 1.9	− 5.3 ± 4.2	~ 1	0.0 ± 2.8	0.1 ± 6.3	1.0
	Female	41.3 ± 2.2	39.0 ± 2.9	40.0 ± 3.2	< 0.001	− 2.3 ± 1.9	− 5.5 ± 4.7	0.205	− 1.3 ± 2.5	− 3.0 ± 6.0	0.4
LPF	Left	42.1 ± 3.4	41.9 ± 2.3	42.5 ± 2.3	~ 1	− 0.3 ± 2.9	− 0.3 ± 6.5	~ 1	0.4 ± 3.3	1.3 ± 7.9	3.0
	Right	40.6 ± 3.2	40.3 ± 2.4	41.1 ± 4.0	~ 1	− 0.4 ± 2.9	− 0.6 ± 7.1	0.914	0.5 ± 2.3	1.1 ± 5.9	2.7
	Male	41.1 ± 3.5	41.4 ± 2.6	41.2 ± 3.2	~ 1	0.3 ± 2.4	1.0 ± 5.9	~ 1	0.1 ± 2.8	0.5 ± 6.9	0.7
	Female	42.4 ± 3.0	40.3 ± 1.6	43.7 ± 2.6	0.096	− 2.0 ± 3.3	− 4.4 ± 7.5	0.297	1.3 ± 2.9	3.4 ± 6.9	1.6
TrPF	Left	44.3 ± 3.4	43.0 ± 3.1	44.4 ± 3.0	0.009	− 1.3 ± 2.2	− 2.8 ± 5.0	~ 1	0.1 ± 1.8	0.3 ± 4.1	1.1
	Right	43.6 ± 2.2	42.5 ± 2.1	44.0 ± 2.6	< 0.001	− 1.1 ± 1.4	− 2.5 ± 3.0	0.554	0.4 ± 1.5	0.9 ± 3.4	1.4
	Male	44.7 ± 2.6	43.6 ± 2.3	44.6 ± 2.5	0.001	− 1.1 ± 1.8	− 2.3 ± 3.9	~ 1	− 0.1 ± 1.3	− 0.1 ± 2.9	0.9
	Female	42.0 ± 2.9	40.4 ± 2.0	43.1 ± 3.4	0.021	− 1.6 ± 2.0	− 3.7 ± 4.8	0.196	1.1 ± 2.1	2.6 ± 5.1	1.7

Difference Rest→21 k: T2 value loss in milliseconds caused by half-marathon running. Difference Rest→Control: T2 value recovery to baseline levels in milliseconds; Detailed Information on ANOVA repeated measures statistics is given in supplementary table in Online Resource 4

*LF* Lateral Femur, *LT* Lateral Tibia, *MF* Medial Femur, *MT* Medial Tibia, *LatC* Lateral Condyle, *MedC* Medial Condyle, *LPF* Lateral Patellofemoral, *TrPF* Troclea Patellofemoral, *PRI* Proportional Recovery Index

^a^Mean ± standard deviation

### T2 values and differences between subgroups (gender and laterality)

In our sample, baseline T2 values were higher in the right knee (43.4 ± 2.3 ms) compared to the left (42.8 ± 2.8 ms) (Table 2). Post-run, the left knee decreased by − 2.0% (p = 0.002) and the right by − 2.1% (p < 0.001). Laterality effects were observed, with the left knee showing significant alterations in LT, MF, MT, MedC and TrPF, and the right knee in MF, MedC, LatC and TrPF (Table 5).

Gender analysis revealed that the baseline T2 values were slightly higher in males (43.6 ± 2.4 ms) than in females (41.5 ± 2.5 ms) (Table 2). Post‐run, males experienced a reduction of − 2.0% (43.6–42.7 ms, p < 0.001), with significant decreases in LF, MF, MT, MedC, LatC and TrPF. Females showed a − 2.1% decrease (41.5–40.6 ms, p = 0.033), with statistically significant reductions limited to MedC and TrPF (Table 5).

## Discussion

This study investigated the effects of a half-marathon on KAC hydration, using a novel dictionary-matching T_2_ method as a biomarker for post-run changes, combined with a structured recovery exercise programme. Post-run KAC T_2_ reductions across multiple knee compartments indicated transient KAC dehydration, with most compartments returning to baseline values after one week. However, incomplete recovery in the LatC and left knee’s MedC, alongside an overcompensatory T_2_ increase in the right MT, may reflect biomechanical adaptations to repetitive loading. For instance, persistent T2 reductions could indicate collagen network remodelling to enhance tensile strength (Mosher et al. 2005), while transient overhydration might facilitate nutrient diffusion during recovery (Coburn et al. 2023b; Coelho et al. 2025). Although we lack direct biomarkers of adaptation (e.g., collagen turnover), the return of global T2 to baseline aligns with prior reports of cartilage resilience after controlled exercise (Luke et al. 2010; Khan et al. 2022).

The observed T2 changes reflect biomechanical stress patterns during running, particularly in load-bearing regions. Pronounced T_2_ changes in several compartments (MF, MT, MedC, LatC) likely reflect their greater role in weight distribution, which reflects alterations in KAC matrix biochemistry. Such changes, if recurrent or inadequately recovered, may lead to long-term structural changes, particularly in individuals with pre-existing conditions, such as post-partial meniscectomy knees (Lindner et al. 2022) where running may increase the risk of damage from excessive loading. Our study showed no significant T_2_ changes in LPF components of healthy individuals, but the trochlear region exhibited a reversible post-run T_2_ decrease, suggesting effective recovery in healthy runners. However, patellofemoral pain syndrome (PFPS) patients may experience compromised recovery in these regions due to biomechanical imbalances and chronic stress, potentially leading to long-term KAC damage (Zaffagnini 2010). This highlights the need for biomechanical interventions and KAC monitoring to optimise training and recovery strategies in at-risk populations. Future research should explore how biomechanical imbalances, such as patellar malalignment, affect T_2_ recovery in PFPS patients compared to healthy runners with different recovery programmes.

Our study used a structured recovery protocol, involving low-intensity running, strength training, and rest, which appeared to be effective in restoring KAC baseline hydration within one week, supporting its utility for detecting compartment-specific stress and guiding biomechanically informed training programmes. Although nutritional factors were uncontrolled, these findings suggest that T_2_ mapping may serve as a valuable biomarker for KAC integrity in endurance athletes. The dictionary-matching T_2_ estimation method, which used an echo-modulated curve analysis rather than conventional mono-exponential fitting, proved its utility in detecting compartment-specific hydration changes. This method’s adaptability across clinical and research settings enhances its utility for longitudinal monitoring, particularly in identifying athletes at risk of overuse injuries.

Previous studies have debated the effect of high-impact activities, like running on KAC health. While some highlight running’s protective role through collagen synthesis (Boxer et al. 2022), others indicate potential damage if not properly monitored (Dreiner et al. 2022). Our findings reconcile these perspectives, showing running-induced dehydrations up to 5% in some sections, particularly in the medial compartments, which reflects clinically relevant changes resembling those observed in the early stages of osteoarthritis, suggesting acute stress and not pathology. Yet its reversibility under structured recovery aligns with evidence demonstrating that controlled exercise promotes cartilage resilience (Luke et al. 2010). Although a 21 km run (half marathon distance) is often considered a moderate-intensity endurance event for trained athletes (when compared to a full marathon), our findings show that the mechanical and biochemical load imposed on KAC is significant. The pronounced T_2_ reductions in some compartments suggest significant changes in KAC hydration and integrity, implying that a 21 km run is, in fact, a sufficiently challenging stimulus. These findings support the notion that even a moderate duration event can trigger relevant physiological responses, which, if not adequately monitored and recovered, may contribute to the predisposition to long-term structural changes, as seen in athletes with high cumulative training loads (Ma et al. 2024).

Individual factors like age, gender, fitness level, and pre-existing conditions significantly influence recovery needs. Right-knee dominant runners showed asymmetrical T2 patterns, likely due to asymmetrical loading, which may lead to distinct recovery patterns or long-term structural changes (Rubin et al. 2021). This phenomenon was also observed in sport-specific cohorts such as basketball players, where seasonal training impacts regional T2 values (Nosrat et al. 2023). Gender-related differences appeared to influence recovery patterns with males showing higher baseline T_2_ values and more efficient recovery in LatC, whereas females showed slightly diminished recovery in certain compartments, with residual T2 reductions. These data suggest potential hormonal influences, such as estrogen, which has been shown to play a protective role in KAC health (Lu et al. 2022), or biomechanical differences. Although previous studies found conflicting results on gender-related KAC T_2_ variations (Macchi et al. 2021; Khandelwal et al. 2022), our results highlight the need to consider these factors in training and rehabilitation protocols. Our sex-based analysis focused on biological differences rather than sociocultural gender identity. While sex-related variations in T2 recovery may reflect estrogen’s chondroprotective effects (Lu et al. 2022), future studies should explore gender-related factors (e.g., training habits, psychosocial stress) that could indirectly influence cartilage health. Such variability emphasizes the need for personalised interventions. Addressing asymmetries through targeted interventions, such as strength training or footwear modifications (Chechik et al. 2021; Rubin et al. 2021), could mitigate KAC damage and promote long-term joint health. Our results also contextualise clinical conditions associated with specific KAC compartments, such as Iliotibial Band Syndrome that affects the lateral compartment (Gupta et al. 2023) or Medial Tibial Stress Syndrome in MT and MF (Lee et al. 2025), both common in long-distance runners. Future research should explore gender-specific factors to allow personalised training and recovery strategies.

This study highlights the importance of balancing training intensity and recovery to prevent KAC OTS, as evidenced by KAC T_2_ recovery patterns. Despite its strengths, some limitations were identified, such as a small sample size, a focus only on asymptomatic athletes, and a lack of control group group (e.g., rest-only) to determine if recovery resulted from the recovery protocol or natural processes. The absence of a standardised pre-scan rest period for the rest and control MRI’s may have introduced variability as participants could have engaged in uncontrolled low-intensity activities prior to scanning. External factors such as running technique, knee force distribution, nutrition and footwear were uncontrolled. Additionally, magic angle effects may have affected certain KAC components, potentially contributing to the extreme T_2_ variations observed. Future research should include the application of the EMC T2 technique in patients with pathological conditions, before and after treatment.

In conclusion, this study showed that half-marathon running induces reversible changes in knee KAC hydration, with recovery occurring within a week coincident with our structured recovery programme. In the absence of a rest‑only control group or pre‑intervention recovery measures, we cannot determine whether this protocol offered an advantage over natural recovery. These findings highlight the importance of the dictionary-matching T2 method to monitor KAC responses to a mechanically demanding stimulus.  Future studies should explore the long-term effects of high-impact activities on KAC and integrate T_2_ mapping with other biomarkers to obtain a more comprehensive understanding of KAC health and performance optimisation.

## Supplementary Information

Below is the link to the electronic supplementary material.

Supplementary file1 (XLSX 11 KB)

Supplementary file2 (XLSX 11 KB)

Supplementary file3 (XLSX 13 KB)

Supplementary file4 (XLSX 15 KB)

## Abbreviations

BMI: Body mass index
EMC: Echo-modulation curves
Ft: Trochlear femur
KAC: Knee articular cartilage
KOSS: Knee injury and osteoarthritis outcome score
LatC: Lateral condyle
LF: Lateral femoral
LFpf: Lateral femur of the PF
LPF: Lateral patellofemoral
LPpf: Lateral patella of the PF
LT: Lateral tibial
MedC: Medial condyle
MESE: Multi-echo spin-echo
MF: Medial femoral
MRI: Magnetic resonance imaging
MT: Medial tibial
OTS: Overtraining syndrome
PF: PatelloFemoral
PFPS: Patellofemoral pain syndrome
PRI: Proportional recovery index
Pt: Trochlear patella
T2Tot: Total T2
TrPF: Trochlear patellofemoral
